# Isolation and Characterization of St-CRPs: Cysteine-Rich Peptides from the Arctic Marine Ascidian *Synoicum turgens*

**DOI:** 10.3390/md24050168

**Published:** 2026-05-08

**Authors:** Ida K. Ø. Hansen, Philip B. Rainsford, Johan Isaksson, Kine Ø. Hansen, Klara Stensvåg, Anastasia Albert, Terje Vasskog, Tor Haug

**Affiliations:** 1 The Norwegian College of Fishery Science, Faculty of Biosciences, Fisheries and Economics, UiT The Arctic University of Norway, Breivika, N-9037 Tromsø, Norway; klara.stensvag@uit.no; 2 Department of Chemistry, Faculty of Science and Technology, UiT The Arctic University of Norway, Breivika, N-9037 Tromsø, Norway; philip.rainsford@sund.ku.dk (P.B.R.); johan.isaksson@uit.no (J.I.); 3 Department of Biomedical Sciences, Faculty of Health Sciences, University of Copenhagen, 2200 Copenhagen, Denmark; 4 Department of Pharmacy, Faculty of Health Sciences, UiT The Arctic University of Norway, Breivika, N-9037 Tromsø, Norway; kine.o.hanssen@uit.no (K.Ø.H.); terje.vasskog@uit.no (T.V.); 5 Norce, Siva Innovasjonssenter, Sykehusveien 21, 9019 Tromsø, Norway

**Keywords:** arctic, ascidian, sea squirt, peptides, cysteine-rich, antimicrobial

## Abstract

Ascidians are a group of marine invertebrates, most of which are sessile and soft-bodied. Their lack of an adaptive immune system makes them rely on innate immune responses to detect and eliminate invading microbes. Antimicrobial peptides (AMPs) play an essential part in this process. In this paper, we present the isolation, structure elucidation, and bioactivities of two new cysteine-rich peptides (CRPs) from the Arctic marine ascidian *Synoicum turgens*. The sequences and structures of the peptides were determined by Edman degradation sequencing, mass spectrometry, and NMR analysis. This revealed two novel 2 kDa peptides, St-CRP-1 and St-CRP-2, with neutral net charge and C-terminal amidation. St-CRP-1 consisted of 18 amino acids and displayed selective and moderate growth inhibition of two Gram-positive bacterial strains (*Bacillus subtilis* and *Corynebacterium glutamicum*) at 24.6 µM, whereas St-CRP-2 consisted of 19 amino acids and inhibited the growth of *B. subtilis* at 49.2 µM. St-CRP-1 had no effect on two mammalian cell lines or the brine shrimp *Artemia salina* at the highest concentration tested. Structural analysis of the St-CRPs indicated a Cys1–Cys6, Cys2–Cys4, and Cys3–Cys5 disulfide connectivity, which is also found in alpha-defensins. The results from this study show that Arctic marine ascidians are a rich source of novel bioactive peptides.

## 1. Introduction

Peptides are ubiquitous natural products, widely abundant and found in all living organisms, from prokaryotes to mammals. Many small peptides (<50 amino acids) are bioactive, displaying various activities such as analgesic, cytotoxic, antihypertensive, antimicrobial, antioxidative, antiviral, and immunoregulatory properties [1]. Many peptides also show high potency and selectivity, as well as low toxicity against normal human cells [2]. Furthermore, most peptides are usually less allergenic compared to larger proteins when administered to mammals [3]. Natural peptides are therefore interesting candidates for pharmaceutical research by serving as templates for developing new therapeutic drugs. There are currently over 80 peptide drugs on the global market, and more than 700 different peptides are in clinical development or in preclinical studies, many of which are derived from natural sources [4].

Antimicrobial peptides (AMPs), also referred to as host defense peptides, are produced by all living organisms, in eukaryotes, as an important part of their innate immune system [5,6]. Due to their natural antimicrobial properties, AMPs are promising candidates for addressing the growing problem of antibiotic-resistant pathogenic bacteria. They are particularly attractive because of their broad-spectrum activity and their relatively low propensity for inducing resistance [7]. One group of diverse AMPs is called defensins. Defensins are a family of cysteine-rich AMPs found in vertebrates, invertebrates, plants, and fungi. They consist of a characteristic β-sheet core structure and are most often stabilized with six disulfide-linked cysteines [8]. Defensins exhibit a broad-spectrum antimicrobial activity against bacteria, fungi, and viruses [9].

While linear peptides show limited promise as both orally and parentally administered drugs because of poor in vivo stability (due to, e.g., proteolytic degradation) and limited membrane permeability [2], cysteine-rich peptides (CRPs) are emerging as a promising class of drug lead candidates and/or templates for drug development [10]. Introduction of disulfide bonds in peptides seems to be among nature’s solutions to the problem of proteolytic degradation. Disulfide bonds effectively constrain peptide topology, resulting in increased structural rigidity and proteolytic resistance [11,12]. Cysteine knot peptides (defined by their three disulfide bridges) and small cysteine-rich proteins are a special sort of peptides containing diverse structures and displaying a wide variety of bioactivities [13].

Marine invertebrates are an increasingly interesting source of novel bioactive peptides because of their ability to thrive in the bacteria-rich environment without the presence of an adaptive immune system [14,15,16]. Ascidians (also known as sea squirts) belong to the phylum of Chordata and the subphylum Urochordata (tunicates), and have been a prolific source of bioactive peptides [14]. A variety of bioactive peptides showing anticancer, antineoplastic, antiviral, antidiabetic, antioxidant, and immunomodulatory properties have been isolated from ascidians. Several of these peptides have been explored as drug candidates, including a few in clinical trials [17].

As part of our ongoing search for novel AMPs from Arctic marine organisms, two novel cysteine-rich AMPs, turgencin A and B, were isolated from the colonial ascidian *Synoicum turgens* [18]. The peptides were 35–36 amino acids in length (3.5–3.7 kDa), containing 3 disulfide bridges with an unusual disulfide connectivity of Cys1–Cys6, Cys2–Cys5, and Cys3–Cys4. During the isolation of these peptides, we recognized a series of 2 kDa peptides in the same extract, with putative antimicrobial properties. Preliminary mass spectrometric analysis indicated the presence of multiple cysteines in these peptides. In this study, two small AMPs (18–19 amino acids in length) having three disulfide bridges were isolated from *S. turgens*. St-CRP-1 was sequenced using Edman degradation and Liquid chromatography–mass spectrometry LC-MS/MS fragmentation, and its structure was confirmed by nuclear magnetic resonance (NMR) analysis. The sequence and structure of St-CRP-2 were determined solely by liquid chromatography tandem-mass spectrometry (LC-MS/MS). This revealed, for both peptides, disulfide connectivity similar to that of alpha-defensins: Cys1–Cys6, Cys2–Cys4, and Cys3–Cys5.

## 2. Results and Discussion

### 2.1. Peptide Purification and Mass Spectrometry Analysis

**Figure 1:**
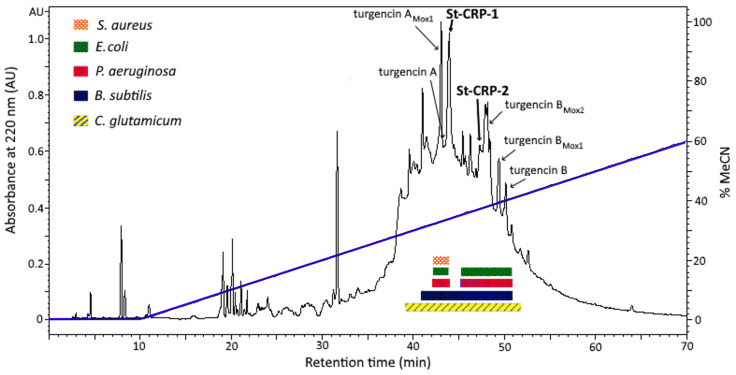
Preparative RP-HPLC-DAD chromatogram (recorded at 220 nm) of the 40% MeCN SPE fraction of *S. turgens*. The peak fractions containing the St-CRPs and the turgencins are marked with arrows. HPLC fractions displaying antibacterial activity are indicated by colored boxes below the chromatogram. The blue line shows the linear gradient (0–60%) of MeCN containing 0.05% TFA.

Colonies of *S. turgens*, collected from the coast of Svalbard, were lyophilized, crunched, and extracted with 60% acidified acetonitrile (MeCN). After removing the sediment, the extract was separated into an organic phase and an aqueous phase (containing a high concentration of salt). The aqueous phase was subjected to solid-phase extraction (SPE) to remove the salt content and to achieve rough-compound separation based on their polarity. Antibacterial screening was performed on the organic phase and the 5 fractions obtained after SPE against a panel of bacterial strains, including the Gram-positives *Corynebacterium glutamicum*, *Bacillus subtilis*, *Staphylococcus aureus*, as well as the Gram-negatives *Escherichia coli* and *Pseudomonas aeruginsa*. All fractions tested displayed antibacterial activity, but the 40% MeCN SPE fraction was the most potent fraction, mainly against the Gram-positive strains *C. glutamicum* and *B. subtilis* (Table S1 in the SI). This fraction was therefore subjected to further fractionation by preparative reversed-phase high-performance liquid chromatography-diode array detector (RP-HPLC-DAD), and the collected one-minute HPLC fractions were tested against the same panel of bacteria as the SPE fractions to identify which fractions/compounds might be causing the antibacterial effect. Such bioassay-guided purification has proven effective when discovering and isolating novel marine AMPs [19,20,21]. Several of the obtained HPLC fractions (fractions 40–52), containing compounds eluting at approximately 30–40% MeCN, displayed antibacterial activity against several of the test strains (Figure 1).

High-resolution mass spectrometry (HR-MS) analysis of the active fractions proved that many of them (fractions 42–43 and 47–51) contained the previously described AMPs turgencin A and B, both also having various oxidized versions (Figure 1) [18]. The turgencins (3.5–3.7 kDa) were originally isolated from the 80% MeCN SPE fraction due to the much higher abundance of these peptides in that SPE fraction [18]. In the present study, HPLC fraction 44 (displaying activity against all test strains), contained only minor amounts of the previously described AMP turgencin A_Mox1_ [18], indicating that another compound or compounds might be responsible for the activity observed. The most abundant molecule in this fraction was a smaller peptide named St-CRP-1. Mass-to-charge (*m/z*) ions recorded for this peptide were *m/z* 1019.8 and *m/z* 680.2, corresponding to [M + 2H]^2+^ and [M + 3H]^3+^, respectively. The monoisotopic mass of St-CRP-1 was determined to be 2037.68 Da by doing deconvolution of the isotopes (Figure S1 in the SI).

Another peptide with a similar size to St-CRP-1 was discovered in the broad-spectrum antibacterial HPLC fraction 48 (Figure 1). This peptide was named St-CRP-2. *m/z* ions recorded for this peptide were *m/z* 1003.9 and *m/z* 669.6, corresponding to [M + 2H]^2+^ and [M + 3H]^3+^, respectively. The monoisotopic mass of St-CRP-2 was determined to be 2005.75 Da (Figure S2 in the SI). However, the most abundant molecule in this active HPLC fraction was the AMP turgencin B_Mox2_. The peaks containing the St-CRPs are marked in bold in the RP-HPLC-DAD chromatogram in Figure 1.

The St-CRPs proved to be challenging to purify, as they coeluted with several other peptides of similar hydrophobicity. Another obstacle was the poor solubility of the isolated peptides after drying. A prolonged process of optimizing the RP-HPLC method yielded sufficient material of St-CRP-1 (1.2 mg) for NMR and bioactivity analysis. The amount of pure St-CRP-2 (0.6 mg) was only sufficient for the antibacterial assays and MS analysis. Ultra-high performance liquid chromatography-DAD-MS (UHPLC-DAD-MS) analysis of the isolated peptides indicated a purity of >95% for both St-CRPs (Figures S3 and S4 in the SI).

### 2.2. Sequence Analysis

Edman degradation analysis of St-CRP-1 revealed an 18-residue N-terminal sequence (CCDQCYGFCRLVDNCCNS). The calculated monoisotopic mass of this sequence, assuming the six cysteines form three disulfide bridges, is 2038.70 Da. The mass difference between the measured and calculated mass of around -1 Da can be explained by a C-terminally amidated serine. C-terminal amidation occurs in all previously sequenced peptides from *S. turgens* [18], and is a known feature in AMPs from eukaryotic organisms [22]. The sequence was confirmed by NMR analysis.

The sequence of St-CRP-2 was obtained by *de novo* sequencing using MS/MS. The peptide was treated with Tris(-carboxyethyl) phosphine (TCEP) at an acidic pH to break the disulfide bonds and subsequently analyzed on a UHPLC-QToF-MS apparatus. This analysis yielded good sequence coverage, providing a 19-residue sequence (SCCEYCSXSCXVSGXXCCQ) with a C-terminally amidated glutamine (Figure S5 in the SI). Four amino acids in the MS/MS spectra were identified as either leucine or isoleucine (both with a monoisotopic mass of 113.08 Da), but the method used could not distinguish between them; hence, the X positions noted in the sequence. With additional material available on the peptide, the precise identity of these amino acid positions could be determined through Edman degradation analysis or genetic characterization. The proposed fragments from MS-product (UCSF, ProteinProspector v.6.4.0, https://prospector.ucsf.edu/prospector/mshome.htm, accessed on 13 October 2022) corresponded to the observed fragments in the MS/MS analysis and confirmed the sequence. The calculated monoisotopic mass of this sequence, assuming three disulfide bridges, C-terminal amidation, and replacing X with leucine, is 2005.75 Da—the same mass as measured by HR-MS.

**Figure 2:**
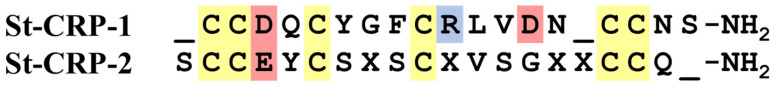
Sequence alignment of St-CRP-1 and St-CRP-2. Gaps (_) are introduced to maximize the alignment. Residues: yellow = Cys; red = acidic amino acids; blue = basic amino acids; X = Ile/Leu.

Sequence alignment of the St-CRPs illustrates the similarities between the two peptides (Figure 2). They share the same cysteine pattern (CC-C-C-CC), are both C-terminally amidated, and neutrally charged with a calculated pI of 6.94 (St-CRP-1) and 6.58 (St-CRP-2). NCBI BLAST (v2.17.0) analyses revealed no sequence similarities to other known peptides or proteins. In addition, no similarities were found to any of the major AMP families present in the CAMP_R3_ database by using the CAMPSign tool [23]. Furthermore, only 193 of the 6309 antimicrobial peptides registered at APD3 have a net charge of 0, of which 38 have structures with three disulfide bonds. Most of these cysteine-rich neutral peptides come from plants (34 out of 38), and their size differs between 26 and 46 amino acids [24].

Knowledge obtained from the structures gave some clues about the solubility obstacles. Prior to this current information, the peptides were dissolved in pure water, but with variable results. The poor aqueous solubility of the St-CRPs is likely a consequence of their neutral net charge and compact disulfide-constrained structure, which together limit solvation and promote exposure of hydrophobic surface patches, thereby favoring peptide aggregation. The solubility of the peptides improved when a small amount of dimethyl sulfoxide (DMSO) was added first, before diluting the DMSO concentration considerably with water. DMSO at high concentrations has been known to interfere with bioassays, and the final concentration needs to be kept at a minimum, with appropriate controls, to avoid false positives [25].

### 2.3. Structure Determination

**Figure 3:**
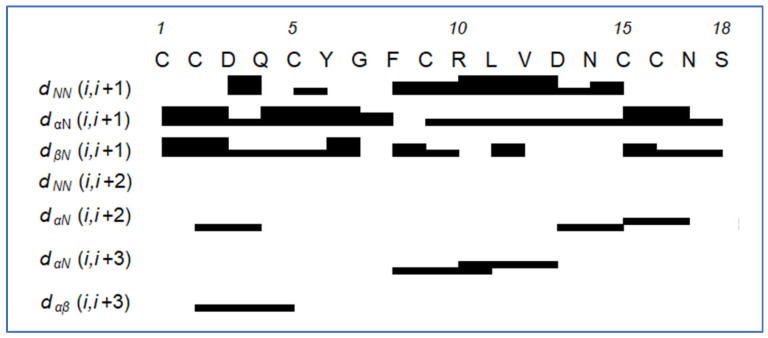
Inter-residual NOEs for St-CRP-1 between adjacent residues extracted from 100, 200, and 300 ms NOESY NMR experiments. The line thickness for the ‘i, i+1’ couplings indicates the strength of the correlation: the thicker the line, the stronger the cross peak. For ‘i, i+2’ and greater, the lines indicate which two residues dipolar couplings can be identified between specified backbone residues. α: proton from C-alpha position; β: proton from C-beta position; N: amide proton.

The water-suppressed ^1^H spectra of St-CRP-1 were clean, with no impurities above 5 mol%, and well-resolved. ^15^N-HSQC (^15^N-heteronuclear single quantum coherence) and TOCSY (total correlation spectroscopy) spectra enabled the unambiguous assignment of all 18 amino acid residues (Tables S2 and S3 in the SI). The sequence was assigned by NOE (nuclear Overhauser effect) hopping supported by high-resolution HMBC (heteronuclear multiple-bond correlation spectroscopy) correlations through the backbone carbonyls where possible. In total, 69 inter-residue backbone–backbone and backbone–sidechain through-space correlations could be extracted from the collected 100, 200, and 400 ms mixing time NOESYs (nuclear Overhauser effect spectroscopy). These NOEs were consistent with the sequence for St-CRP-1 (Figure 3).

An additional 144 non-sequential inter- and intra-residual NOEs were extracted, for a combined total of 213 unique NOEs. The NOEs were qualitatively classified into four categories—Strong, Medium, Weak, Very Weak—based on their intensities and correspond to upper limit distance constraints of 2.7, 3.5, 5.0, and 6.0 Å, respectively.

**Figure 4:**
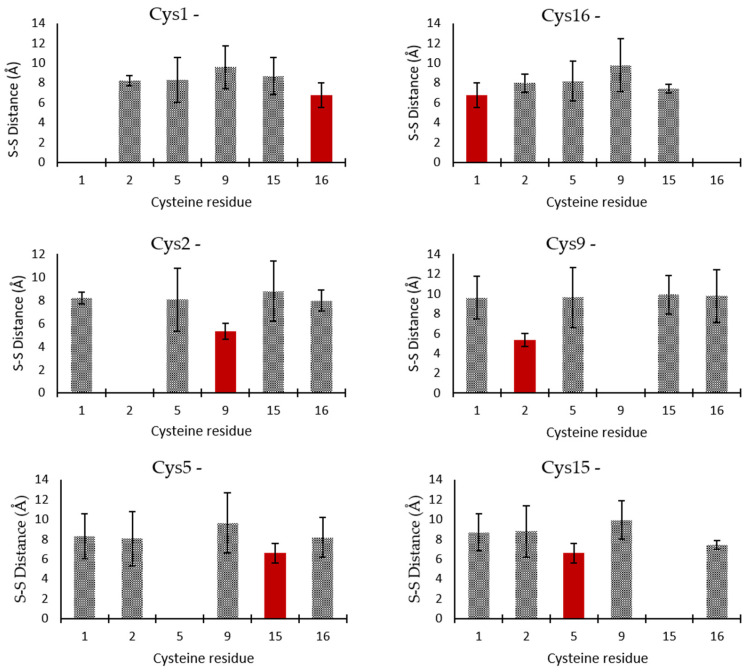
Average inter-sulfur distances for the six cysteines identified in St-CRP-1. The shortest distance is highlighted in red, being consistent with a C1–C6/C2–C4/C3–C5 disulfide pattern.

Three-dimensional structures were generated by simulated annealing protocols to produce a series of energetically minimized structures. First, structures were generated from an extended chain without any designation of disulfide bonds, applying only distance constraints to fold the peptide. Three iterations were calculated, during which any violations of interatomic distances due to overlaps or other sources of erroneous input were resolved to refine the fold. A batch production run of 500 structures was generated using the iterated constraints, and the 10 most energetically favorable structures were selected. The sulfur–sulfur interatomic distances were plotted (Figure 4). By comparing the distances between each cysteine sulfur, the nearest and therefore most likely bonding partners were identified. The determined disulfide bridge partners were Cys1–Cys16, Cys2–Cys9, Cys5–Cys15, giving a C1–C6/C2–C4/C3–C5 disulfide pattern.

**Figure 5:**
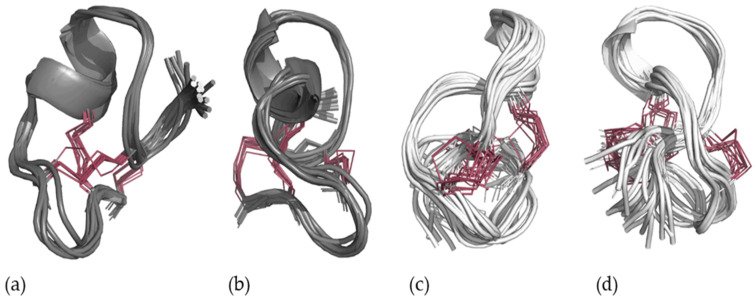
The 19 lowest energy structure ensembles generated from the simulated annealing of St-CRP-1 with the defined disulfide bridge pattern of C1–C6/C2–C4/C3–C5 (highlighted in red) in combination with NOE constraints and TALOS+ predicted dihedrals (**a**), and the 90-degree rotated view (**b**), compared to the knot structure (**c**) and its rotated view (**d**).

A final production batch was calculated with the C1–C6/C2–C4/C3–C5 disulfide pattern, using the same simulated annealing protocol together with the refined distance constraints, adding also dihedral bond angle constraints predicted from the H, N, C, CA, CB, HA, and HB chemical shifts using TALOS+. The lowest-energy structures (energies below 2 kcal) were selected for analysis, representing 38 of the 500 structures. Evaluation of the structures revealed that the structures adopt one of two energetically equivalent conformations—an open fold with a small stretch of helix (Figure 5a,b—21/38 structures), and a knot conformation (Figure 5c,d—17/38 structures).

### 2.4. Knot or Not a Knot?

**Figure 6:**
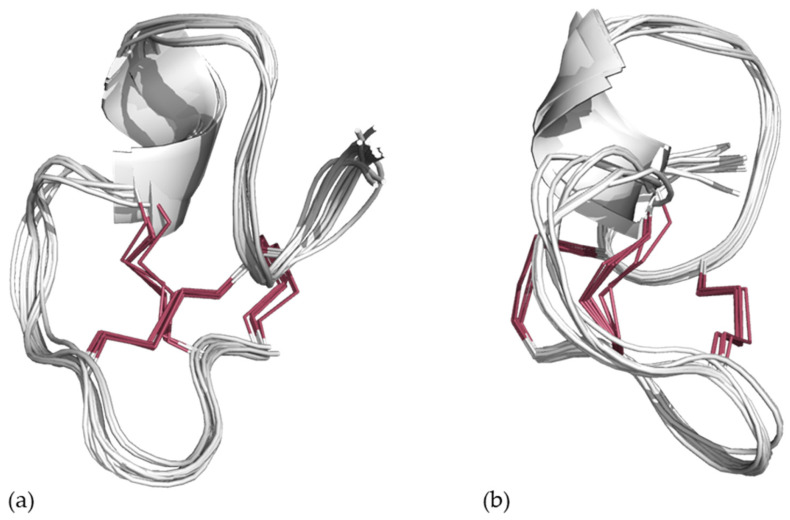
The 19 lowest energy structure ensembles generated from the simulated annealing of St-CRP-1 with the defined disulfide bridge pattern of C1–C6/C2–C4/C3–C5 (highlighted in red) in combination with NOE constraints and TALOS+ predicted dihedrals (**a**), and the 90-degree rotated view (**b**).

Both these structures satisfy the experimental constraints equally well. The structures were evaluated for correlations that would be expected according to the conformation adopted but were absent in the data set, indicating if one conformation is favored by the acquired data. The knot structure is more condensed, and if this conformation were populated, one would expect to observe a range of correlations between the C-terminus and residues 6–9 where the knot is formed. The clearest example was between HA-Tyr6 and HA-Cys16 (a distance of 4 Å). Since this correlation was not observed in the data and no clear inconsistencies with the open conformation could be found, we introduced a repulsion between HA Tyr6 and HA Cys16 and recalculated the structures. This abolished the knot conformation and resulted in a final structure ensemble presented in Figure 6. Three out of the 31 lowest energy structures had a backbone root mean square deviation (RMSD) of more than 2.5 Å from the lowest energy structure, and these were omitted from the graphical representation as a minor outlying conformation for visual clarity. The backbone RMSD of the other 28 structures was all 1.0 Å or less (Table S4 in the SI).

A short alpha-helical loop stretches between Cys9 and Val12, which could be identified and was amply represented in the calculated structure ensemble (15 out of 19 in the final ensemble). Strong NH(i)-NH(i + 1), and medium strength αH(i)- NH(i + 1) NOEs were recorded for this stretch, which is consistent with an alpha-helical conformation being populated. Furthermore, two NH(i)-NH(i + 3) correlations were also identified from residues 8 to 11 and 10 to 13, which is consistent with an alpha helix.

The disulfide bridge pattern for both peptides was confirmed by LC-MS/MS using a sequential alkylation method, introduced by Albert et al. [26]. The peptides were reduced and alkylated with different maleimides on a solid phase before sequencing. The reduction and alkylation process resulted in a mixture of different alkylation patterns where the number of cysteines with different alkylating agents was 2 × NMM + 4 × NEM, 4 × NMM + 2 × NEM, 2 × NMM + 4 × NCM, 4 × NMM + 2 × NCM, 2 × NEM + 4 × NCM, 4 × NEM + 2 × NCM, 2 × NMM + 2 × NEM + 2 × NCM, 2 × NMM, and 4 × NMM without further alkylation, and 6xNMM for both peptides. In addition, St-CRP-1 showed a pattern of 2 × NMM + 2 × NEM, without further reduction and alkylation, which was not seen for St-CRP-2. Several of the alkylation patterns could be used to determine the disulfide connectivity, but the most convenient pattern was the 2 × NMM + 2 × NEM + 2 × NCM, where each bridge results in a pair of cysteines with the same alkylating agent. The other alkylating patterns were used to confirm the findings from this pattern. Also, N-terminal acetylation was observed following reduction and alkylation of the peptides. This is not a native post-translational modification but most likely results from a side reaction during sample preparation. Due to the quality of these MS/MS spectra, they were used to analyze cysteine connectivity.

**Figure 7:**
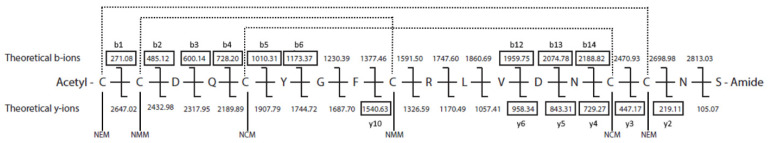
The alkylation pattern 2 × NEM + 2 × NMM + 2 × NCM of the acetylated doubly charged [M + 2H]^2+^ molecular ion (*m/z* = 1459.05) of St-CRP-1. The framed masses correspond to b- and y-ions identified in the MS/MS spectra. The dotted lines illustrate the disulfide bridges.

**Figure 8:**
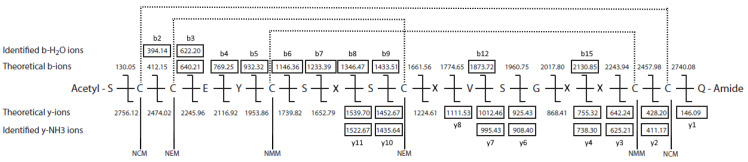
The alkylation pattern 2 × NCM + 2 × NEM + 2 × NMM of the acetylated doubly charged [M + 2H]^2+^ molecular ion (*m/z* = 1443.09) of St-CRP-2. The framed masses correspond to b-, b-H_2_O, y-, and y-NH_3_ ions identified in the MS/MS spectra. The dotted lines illustrate the disulfide bridges. The X in the sequence is either I or L.

To determine cysteine connectivity by MS/MS analysis, the [M + 2H]^2+^ ion and the corresponding acetylated ion were used for both peptides. The *m/z* value of this ion differs depending on the alkylation pattern, but for the 2 × NMM + 2 × NEM + 2 × NCM pattern, the St-CRP-1 peptide gave *m/z* = 1438.04 (acetylated *m/z* = 1459.05, Figure 7) and the St-CRP-2 peptide gave *m/z* = 1422.08 (acetylated *m/z* = 1443.09, Figure 8). The observed b- and y-ions of the acetylated [M + 2H]^2+^ of St-CRP-1 indicate that Cys1 and Cys16 are alkylated with maleimide NEM, Cys2 and Cys9 with NMM, and Cys5 and Cys15 with NCM (Figure 7). This verifies the C1–C6/C2–C4/C3–C5 connectivity for St-CRP-1 obtained by NMR.

St-CRP-2 showed the same disulfide bridge pattern as St-CRP-1. From the acetylated [M + 2H]^2+^ ion of St-CRP-2, two spectra showed the 2 × NMM + 2 × NEM + 2 × NCM pattern. The observed b-ions, b-ions with water loss, y-ions, and y-ions with ammonia loss identified Cys2 and Cys18 to be alkylated with maleimide NCM, Cys3 and Cys10 with NEM, and Cys6 and Cys17 with NMM (Figure 8).

The same cysteine connectivity was confirmed in another spectrum of St-CRP-2, where the identified fragments showed Cys2 and Cys18 to be alkylated with maleimide NEM, Cys3 and Cys10 with NMM, and Cys6 and Cys17 with NCM (Figure S6 in the SI). This gave the same cysteine pattern as for St-CRP-1, a C1–C6/C2–C4/C3–C5 connectivity. Both St-CRPs share key features with previously reported turgencins [18], including the presence of six cysteine residues and a C-terminal amidation. However, they differ markedly in their primary structure, being significantly shorter (18–19 residues vs. 35–36 residues) and containing two pairs of adjacent cysteines, a feature not observed in turgencin A or B. Furthermore, the peptides lack the lysine-rich motifs characteristic of turgencins. These differences suggest that, while originating from the same organism and sharing a cysteine-rich framework, the peptide represents a structurally distinct class of disulfide-stabilized antimicrobial peptides rather than a truncated turgencin analog. In addition, the turgencins have a C1–C6/C2–C5/C3–C4 connectivity. The St-CRPs share the same cysteine connectivity as mammalian alpha-defensins [8], and other AMPs such as aurelin from the jellyfish *Aurelia aurita* [27] and damicornin from the coral *Pocillopora damicornis* [28]. Other than the cysteine connectivity, these peptides share few similarities with the St-CRPs. They are all cationic peptides (damicornin with as many as nine charges) and bigger in size (>30 amino acids), while the St-CRPs have a neutral net charge and fewer than 20 amino acids.

Another peptide family with C1–C6/C2–C4/C3–C5 connectivity is the M2 family of conotoxins. In addition, the majority of the entire M family share the same cysteine pattern (CC-C-C-CC) as the St-CRPs [29]. These cysteine pattern similarities are interesting, but other than that, no relations can be drawn between the M2-family and the St-CRPs based on the information that is available. Many conotoxin families are well described in the literature, but there are limited published data on the biological targets and mechanisms of action of peptides from the M2 branch. It has been reported that some of these peptides induce strong excitatory behavior in mice [29].

A comparison of the identified St-CRPs against peptides within the AMP databases APD3 [30], DRAMP [31], and DBAASP [32], revealed no closely related sequences or structural homologs. While these peptides belong to the general class of cysteine-rich AMPs, their unique combination of short length (18–19 residues), six cysteine residues, and two pairs of adjacent cysteines is highly unusual and not characteristic of established AMP families such as defensins, protegrins, or cyclotides. Moreover, their cysteine spacing and experimentally determined disulfide connectivity do not align with known motifs in these databases, suggesting that these peptides represent a previously uncharacterized structural subclass of disulfide-stabilized AMPs.

### 2.5. Biological Activity

As the HPLC fractions containing the St-CRPs exhibited antibacterial activity (Figure 1), the purified peptides were tested against the same panel of bacteria to verify the antibacterial activity. In addition, St-CRP-1 was tested for toxicity against the brine shrimp *Artemia salina*, and for cytotoxic activity against the human melanoma cancer cell line A2058, and the non-malignant human fibroblast cell line MRC-5. The St-CRPs showed only moderate activity against the bacterial strains tested. St-CRP-1 displayed a minimum inhibitory concentration (MIC) value of 50 µg/mL (24.6 µM) against *C. glutamicum* and *B. subtilis*, whereas St-CRP-2 displayed a MIC value of 100 µg/mL (49.2 µM) against *B. subtilis* (Table 1). None of the peptides were active against the Gram-negative bacterial strains at the highest concentration tested (100 µg/mL), which equals 49.1 µM for St-CRP-1 and 49.2 µM for St-CRP-2, respectively. Also, St-CRP-1 showed no activity in any of the toxicity assays at the highest concentration tested. This conforms well with other neutral CRPs listed in the APD3 database, of which half of these peptides have unknown bioactivity [30]. In some cases, like with the macrocyclic varv peptides from the plant *Viola arvensis*, the plant produces several neutral CRPs with both known bioactivity (varv peptide A—anticancer, and varv peptide E—antiviral and hemolytic) and unknown bioactivity (varv peptide C and D) [33,34].

Compared to some of the turgencins [18], which in general showed much higher antibacterial activity against the same panel of bacteria, one could assume that the main function of the St-CRPs is not to interfere with (inhibit the growth of or kill) bacteria directly. Here, we have tested purified peptides alone in vitro against standard laboratory bacteria. It is plausible that the peptides would be more potent against marine pathogenic bacteria, which are a bigger threat to the animal than the terrestrial strains tested. This has been observed in other studies of marine-derived antimicrobial peptides [20]. However, the peptides might also have other host-defense functions in vivo. Perhaps the St-CRPs generate a synergistic effect together with the turgencins or other compounds from the ascidian. The HPLC-fraction containing St-CRP-1 was active against all bacteria tested, and the fraction containing St-CRP-2 was active against 4 out of 5 strains tested. The St-CRPs were also the dominant compounds within their respective HPLC fractions. Since the isolated St-CRPs showed no activity at 100 µg/mL against the tested Gram-negative strains (*E. coli* and *P. aeruginosa*) or against *S. aureus*, the activity observed in the HPLC fractions may be attributable to other compounds, such as turgencins, or to a synergistic effect between the St-CRPs and co-eluting compounds. Many organisms produce cocktails of different AMPs to defend their survival, and the main functions of many of these peptides are yet to be explored [34].

## 3. Materials and Methods

### 3.1. Materials

The colonial sea squirt *S. turgens* (Phipps, 1774) was collected off the coast of Svalbard in August 2016 (79°33′ N, 18°37′ E) by divers at 20–30 m depth. The sample was identified by Robert A. Johansen, Marbank, Norway (https://www.hi.no/hi/forskning/forskningsgrupper/marbank, accessed on 22 May 2024), and subsequently frozen at −20 °C at sea. The biomass was lyophilized and kept frozen until further processing.

### 3.2. Extraction

Lyophilized samples of the ascidian (100 g) were pulverized and extracted with 5 volumes (*v/w*) of 60% MeCN (HPLC-grade, Sigma-Aldrich, Steinheim, Germany) containing 0.1% trifluoroacetic acid (TFA, HPLC-grade, Sigma-Aldrich, Milford, MO, USA) dissolved in Milli-Q H_2_O (Millipore, Burlington, MA, USA) for 24 h at 4 °C. The mixture was centrifuged, and the supernatant was collected and stored at 4 °C before the residue was extracted once more under the same conditions. Supernatants were pooled and incubated at −20 °C for 1–2 h, causing the formation of two liquid phases, an organic MeCN-rich phase and an aqueous salt-rich phase. The aqueous phase was dried in a ScanSpeed 40 vacuum centrifuge (Labogene ApS, Lillerød, Denmark) and afterward dissolved in 0.05% TFA/Milli-Q H_2_O (*v/v*) to a concentration of 100 mg/mL. To remove salt from the sample, solid phase extraction (SPE) was performed using reversed-phase C18 35 cc Sep-Pak Vac cartridges (Waters, Milford, MA, USA), as described by Haug et al. [35] with some modifications. Briefly, the cartridge was conditioned in MeCN and equilibrated with 0.05% TFA/H_2_O (*v/v*) before the aqueous phase was added. After washing the loaded extract with acidified water, a five-step elution was done with 10, 20, 30, 40, and 80% (*v/v*) MeCN containing 0.05% TFA *(v/v*). The collected SPE eluates were dried in a ScanSpeed 40 vacuum centrifuge and kept frozen at −20 °C until further analysis.

The SPE fractions were resuspended in Milli-Q H_2_O to a concentration of 10 mg/mL. Non-dissolved material was removed by centrifugation, and the supernatant was tested for antibacterial activity.

### 3.3. Peptide Purification and MS Characterization

Active SPE fractions were submitted to purification by RP-HPLC. The separation was performed using an Agilent 218 Preparative gradient LC system coupled to an Agilent 1260 infinity DAD and an Agilent 440-LC fraction collector (Matriks, Oslo, Norway). The column used was an XBridge BEH C18 Prep column (10 × 250 mm, 5 µm, Waters. MA, USA). The mobile phase consisted of A: H_2_O with 0.05% TFA and B: MeCN with 0.05% TFA, where the method was set to run mobile phase A for 10 min, then a gradient of 0–60% of mobile phase B from 10 to 70 min, with a flow rate of 6 mL/min. One-minute fractions were collected throughout the analysis, vacuum dried separately, and redissolved in 500 µL Milli-Q H_2_O before testing for antibacterial activity. All SPE fractions and the active HPLC fractions were submitted to HR-MS analysis, using an Agilent 1290 Infinity UHPLC-DAD system and an Agilent 6540B quadrupole time-of-flight (Q-ToF) mass spectrometer coupled with a dual electrospray ionization (ESI) source. The data was acquired and analyzed by using the Agilent MassHunter softwares (Data Acquisition v.B.06.01, SP1, and Qualitative Analysis v.B.07.00, SP2) (all instruments and software were from Matriks). A standard method was used, running a gradient from 5 to 100% MeCN with 0.1% formic acid (FA) over 8 min with a flow rate of 0.3 mL/min. The separation was done using an Agilent Zorbax Eclipse Plus C18 column (2.1 × 50 mm, 1.8 µm, Matriks, Oslo, Norway).

The HR-MS analysis confirmed the presence of the small (ca. 2 kDa) peptides in some of the antibacterial HPLC fractions derived from the 40% MeCN SPE fraction. To isolate these peptides, the SPE fraction was repeatedly injected on the preparative RP-HPLC system, using an optimized RP-HPLC method. The mobile phase consisted of the same constituents as described above; however, elution was performed by running 20% mobile phase B for 5 min, then a gradient of 20–45% mobile phase B from 5 to 35 min, with a flow rate of 6 mL/min. The peptides were isolated by triggering collection at predetermined timepoints during the run. Each fraction was analyzed using the Agilent HR-MS system, and fractions containing pure peptides were pooled, lyophilized, and kept frozen at −20 °C until further analysis.

### 3.4. Peptide Sequencing

Primary structure determination of St-CRP-1 was performed with Edman degradation sequencing at Eurosequence (Groningen, the Netherlands). For *de novo* MS sequencing of St-CRP-2, 2 µL of 0.5 mM peptide was added to 20 µL of 0.1 M TCEP (Sigma-Aldrich, St. Louis, MO, USA) and 50 µL of 1 mM ammonium formate buffer adjusted to pH 3 with formic acid. The solution was incubated at room temperature for one hour for full reduction of the peptide. The reduced peptide was analyzed on an Acquity I-class UPLC with a Waters Xevo QToF G2 mass spectrometer (Waters, Milford, MA, USA). The separation was performed using an Acquity BEH C18 column (2.1 × 100 mm, 1.7 µm, Waters), and a mobile phase gradient consisting of A: water + 0.1% FA and B: MeCN + 0.1% FA. Fragmentation spectra were obtained by CID fragmentation with a collision energy ramp of 20–50 eV. The fragment spectra provided full coverage of the peptide sequence, and, for confirmation, the proposed sequence was inserted into the MS-product from UCSF (https://prospector.ucsf.edu/prospector/cgi-bin/msform.cgi?form=msproduct, accessed on 13 October 2022) to induce peptide fragmentation. Isoelectric points (pI) were calculated using Innovagen’s peptide property calculator app (https://www.innovagen.com, accessed on 13 October 2022). Sequence similarity searches were performed using the Basic Local Alignment Search Tool (BLAST, https://blast.ncbi.nlm.nih.gov/Blast.cgi, accessed on 14 September 2022), provided by the National Center for Biotechnology Information (NCBI).

### 3.5. NMR Spectroscopy and Calculations

NMR experiments were acquired on an Avance III HD spectrometer equipped with an inverse four-channel probe with cryogenic enhancement for ^1^H, ^2^H, and ^13^C (TCI) operating at 600 MHz for ^1^H (Bruker Biospin, Fällanden, Switzerland).

The sample of St-CRP-1 was prepared by dissolving 0.8 mg of material in 120 μL of H_2_O/D_2_O solution (95/5) in a D_2_O-matched 3 mm Shigemi tube. The following experiments were acquired for the elucidation of St-CRP-1: ^1^H (excitation sculpting), ^13^C, ^15^N-HSQC, ^13^C-HSQC, HMBC (including selective carbonyl HMBC), HSQCTOCSY (80 ms DIPSI), NOESY (100, 200, 300 ms mixing time), ROESY (100 ms spinlock), DQF-COSY, E.COSY, and TOCSY (60, 100 ms DIPSI). Where applicable, gradient-selection and adiabatic pulse sequences were used. Acquisition and processing were done in Topspin 3.5pl7 using standard pulse sequences (Bruker Biospin). Spectral assignment and integration were done in CARA 1.8.4.2.

Starting structures were created as extended chains and folded using a standard simulated annealing protocol (2000 K, 20,000 cooling steps *in vacuo*) based on observed NMR parameters and the absence of disulfide connectivity. Low-energy folds from the previous step were used to generate disulfide-connected starting structures for the final refinements. Finally, production runs of 500 cycles of simulated annealing generated the reported structure ensemble. Structures were generated using XPLOR-NIH 2.52, and secondary structure prediction was made in TALOS+ (https://spin.niddk.nih.gov/NMRPipe/talos/, accessed on 18 August 2022). The NMR data is available at the Biological Magnetic Resonance Data Bank (https://bmrb.io, accessed on 5 November 2020) under accession number 50547.

### 3.6. Reduction and Alkylation of the Peptides

To determine the disulfide connectivity in the peptides, a reduction and alkylation method by Albert et al. was employed [26]. All chemicals used in this method were purchased from Sigma-Aldrich. The protocols for St-CRP-1 and St-CRP-2 were optimized individually, using the described method as a template. An overview of the analytical method and details on the reduction and alkylation procedures will be given here.

St-CRP-1: The SPE column (Empore C18, 3M, St. Paul, MN, USA) was activated with 250 µL MeCN and subsequently equilibrated with 500 µL ammonium formate buffer (50 mM, pH 3). The peptide was dissolved in the same buffer to a concentration of 0.5 mM, and a volume of 500 µL was applied to the column. A volume of 100 µL 0.1 M TCEP was loaded onto the column to selectively reduce available cysteine bridges, and the mixture on the column was incubated for 1 min before the column was washed 3 times with 300 µL of ammonium formate buffer/MeCN 90:10 (*v/v*), and once with 250 µL of the same buffer. Immediately thereafter, the peptide was alkylated by adding 20 µL of 0.5 M *N*-methylmaleimide (NMM) dissolved in buffer, and the solution was left to incubate for 1 h. The sample was eluted from the column with 300 µL 80% MeCN, and MeCN was removed under a gentle stream of nitrogen at 55 °C. To remove excess NMM, 100 µL of 0.5 M thiosalicylic acid (TA) was added and left to react with the remaining NMM for 30 min. The sample was loaded onto a freshly equilibrated SPE column and washed 3 times with 300 µL 10% MeCN and once with buffer. For the second reduction, 100 µL of 0.1 M TCEP was again added and left to incubate for 1 min before the column was washed 3 times with 300 µL 10% MeCN and once with 300 µL buffer. The peptides were alkylated for the second time by adding 20 µL of a 0.5 M solution of *N*-ethylmaleimide (NEM), and the solution was left to incubate for 1 h before the column was washed, as described above. Excess NEM was removed with TA as described for NMM, and after washing and eluting from the column, 20 µL of 0.1 M TCEP was added to the solution and left to incubate for 1 h. The final alkylation was performed by adding 20 µL 0.12 M *N*-cyclohexyl maleimide (NCM), and the solution was left to incubate for 3 h.

St-CRP-2: In general, the same alkylation protocol was used for St-CRP-2, but with some modifications. The peptide was dissolved in the same buffer, but 450 µL of a 0.5 mM peptide solution was added to the column. A volume of 50 µL 0.1 M TCEP was added to the column to selectively reduce available cysteine bridges. After incubation, the column was washed with 500 µL 20% MeCN. Immediately afterward, 10 µL of a 0.5 M NMM solution was added to alkylate the reduced cysteines, and the solution was incubated for 1 h. To remove excess NMM, the column was washed 5 times with 500 µL 20% MeCN. The second reduction and alkylation were performed by adding 50 µL of 0.1 M TCEP to the peptide solution before incubation for 1 min. The column was then washed with 500 µL 20% MeCN and immediately loaded with 10 µL 0.5 M NEM, which was left to incubate for 1 h. A volume of 50 µL 0.5 M TA was added to the column and left to incubate for 0.5 h to react with excess NEM before the column was washed 3 times with 500 µL 20% MeCN. The third and final reduction and alkylation was performed in solution by eluting the peptide from the column with 300 µL 80% MeCN before 20 µL 0.1 M TCEP was added and left to incubate for 1 h to reduce the remaining disulfide bridges. Then, 20 µL of a 0.12 M NCM solution was added to complete alkylation of the last cysteines.

Reduced and alkylated peptides were analyzed using the same MS instrument, column, and mobile phase as described in the sequence analysis method. Mass spectrometric identification parameters were similar as for Albert et al. [26]. For both peptides, a collision energy ramp of 26–58 eV was used for optimal fragmentation.

### 3.7. Antibacterial Activity Assay

SPE fractions and HPLC fractions, as well as the isolated St-CRPs, were screened for activity against the Gram-negative bacteria *E. coli* (ATCC 25922) and *P. aeruginosa* (ATCC 27853), and the Gram-positive bacteria *S. aureus* (ATCC 9144), *C. glutamicum* (ATCC 13032), and *B. subtilis* (ATCC 23857). All isolates were grown in Mueller–Hinton (MH) broth (Difco Laboratories, Detroit, MI, USA) at room temperature. The assays were performed in 96 microwell plates (Thermo Fisher Scientific, Roskilde, Denmark), as previously described [18]. In short, 50 µL of a bacterial dilution containing 1250–1500 bacterial cells were added to each well in a plate preloaded with 50 µL of either SPE in a dilution series, HPLC fractions, or a dilution series of St-CRPs and controls. The purified peptides (>95% purity based on UHPLC-DAD-MS analysis; Figures S3 and S4 in the SI) were dissolved in DMSO (Sigma-Aldrich), vortexed, and diluted with MQ-H_2_O to obtain a stock solution of 500 µg/mL containing 2.5% DMSO. The stock solution was diluted in MQ-H_2_O to obtain final test concentrations ranging from 2.5 to 100 µg/mL. Oxytetracycline hydrochloride (Sigma-Aldrich) was used as a positive (antibacterial) control (0.04–40 µM), MQ-H_2_O as a negative (growth) control, and a DMSO control was made using the highest tested concentration of DMSO (0.25% DMSO). All experiments were done in technical triplicate. Bacterial growth at 35 °C was monitored with an EnVision Multilabel Reader (PerkinElmer, Llantrisant, United Kingdom), where the optical density (OD_595_) was measured every hour for 24 h. The MIC was defined as the concentration resulting in >90% reduction in OD_595_ after 24 h compared to the negative (bacterial growth) control.

### 3.8. Human Cell Viability Assay

The cytotoxic activity of St-CRP-1 was tested on two cell lines: A2058 (a human melanoma cancer cell line, ATCC CRL-11147TM) and MRC-5 (a non-malignant human fibroblast cell line, ATCC CCL-171). The peptides were assayed using a two-fold dilution series, ranging from 5 to 100 µg/mL. The assays were performed as previously described [36]. Cell viability calculation: cell survival (%) = (absorbance treated wells − absorbance positive control)/(absorbance negative control − absorbance positive control) × 100. Both assays were performed in technical triplicate in two independent experiments.

### 3.9. Brine Shrimp Lethality Assay

St-CRP-1 was tested for toxic effect against Artemia salina nauplii as previously described by Haug et al. [37], with some modifications. Sterile filtered (0.22 µm) seawater was added to an illuminated Petri dish with a teaspoon of dried brine shrimp eggs and incubated at 22–24 °C. After 48 h of incubation, 100 µL of seawater containing 10–20 freshly hatched nauplii was added to separate wells in 96 microwell plates (Thermo Fisher Scientific, Roskilde, Denmark). Three dilutions of the peptide were added to the wells (in duplicates) at final concentrations of 100, 50, and 25 µg/mL. The plates were incubated with illumination at 22–24 °C, and dead nauplii were counted after 6 (acute toxicity) and 24 h (chronic toxicity). MQ-H_2_O was used as a negative control, and potassium dichromate (K_2_Cr_2_O_7_, Sigma-Aldrich, St. Louis, MO, USA, 10–1000 ppm) was used as a positive control.

## 4. Conclusions

The worldwide spread of antibiotic resistance has fueled the search for and discovery of novel antibacterial molecules. AMPs are promising candidates because of their broad-spectrum antimicrobial properties and fewer cases of antimicrobial resistance developed towards them. In addition, cysteine-rich AMPs (or CRPs) are generally also less prone to proteolytic degradation. In the present study, two novel cystine-rich peptides, St-CRP-1 and St-CRP-2, were isolated from the Arctic ascidian, *S. turgens*. The peptides consist of 18–19 amino acids, are neutrally charged, and share the same cysteine connectivity as alpha-defensins and the M2 family of conotoxins: C1–C6/C2–C4/C3–C5. A gene characterization of the St-CRPs could reveal the evolutionary relationship between them and other CRPs. The St-CRPs showed selective, moderate antibacterial activity and no cytotoxicity against mammalian cells. Ascidians have proven to be a promising resource for finding novel peptides—potential templates for drug development.

## Figures and Tables

**Table 1:** Bioactivity of St-CRPs.

	Antimicrobial Activity (MIC; μg/mL)					Brine Shrimp Toxicity (LC_50_; μg/mL)	Cytotoxic Activity (IC_50_; μg/mL)	
Peptide	Cg	Bs	Sa	Ec	Pa	As	A2058	MRC-5
St-CRP-1	50	50	>100	>100	>100	>100	>100	>100
St-CRP-2	>100	100	>100	>100	>100	N.t	N.t	N.t

Cg—*Corynebacterium glutamicum*, Bs—*Bacillus subtilis*, Sa—*Staphylococcus aureus*, Ec—*Escherichia coli*, Pa—*Pseudomonas aeruginsa*, As—*Artemia salina*. N.t: Not tested.

## Data Availability

The original contributions presented in this study are included in the article and Supplementary Materials. Further inquiries can be directed to the corresponding authors.

## Supplementary Materials

The following supporting information can be downloaded at: https://www.mdpi.com/article/10.3390/md24050168/s1. Figure S1: MS spectra and deconvoluted MS spectra of St-CRP-1; Figure S2: MS spectra and deconvoluted MS spectra of St-CRP-2; Figure S3: UPLC-PDA chromatogram of St-CRP-1; Figure S4: UPLC-PDA chromatogram of St-CRP-2; Figure S5: De novo sequencing of St-CRP-2; Figure S6: The alkylation pattern 2 × NEM + 2 × NMM + 2 × NCM of the acetylated [M + 2H]^2+^ of St-CRP-2; Table S1: Antimicrobial activity of solid phase extract (SPE) fractions and the organic extract of *S. turgens*; Table S2: Proton (^1^H) NMR and chemical shift assignments for St-CRP-1; Table S3: Carbon (^13^C) NMR and chemical shift assignments for St-CRP-1; Table S4: RMSD of top St-CRP-1 structures generated through final simulated annellation constraints.

## Abbreviations

The following abbreviations are used in this manuscript:

AMPs	Antimicrobial peptides
CRPs	Cysteine-rich peptides
COSY	Correlation spectroscopy
Da	Dalton
DAD	Diode array detector
DMSO	Dimethyl sulfoxide
FA	Formic acid
HMBC	Heteronuclear multiple-bond correlation spectroscopy
HSQC	Heteronuclear single quantum coherence
HR-MS	High-resolution mass spectrometry
K_2_Cr_2_O_7_	Potassium dichromate
LC-MS/MS	Liquid chromatography tandem-mass spectrometry
MeCN	Acetonitrile
MIC	Minimum inhibitory concentration
*m/z*	Mass-to-charge
NCM	*N*-cyclohexyl maleimide
NEM	*N*-ethylmaleimide
NMM	*N*-methylmaleimide
NMR	Nuclear magnetic resonance
NOE	Nuclear Overhauser effect
NOESY	Nuclear Overhauser effect spectroscopy
pI	Isoelectric point
RMSD	Root mean square deviation
ROESY	Rotating-frame nuclear Overhauser effect spectroscopy
RP-HPLC	Reversed-phase high-performance liquid chromatography
SPE	Solid phase extraction
TA	Thiosalicylic acid
TCEP	Tris(-carboxyethyl) phosphine
TFA	Trifluoroacetic acid
TOCSY	Total correlation spectroscopy
UHPLC	Ultra-high performance liquid chromatography
Q-ToF	Quadrupole time-of-flight

## References

[1] Shahidi F., Saeid A. (2025). Bioactivity of marine-derived peptides and proteins: A review. Mar. Drugs.

[2] Fosgerau K., Hoffmann T. (2015). Peptide therapeutics: Current status and future directions. Drug Discov. Today.

[3] Günal-Köroğlu D., Karabulut G., Ozkan G., Yılmaz H., Gültekin-Subaşı B., Capanoglu E. (2025). Allergenicity of alternative proteins: Reduction mechanisms and processing strategies. J. Agric. Food Chem..

[4] Liu M., Svirskis D., Proft T., Loh J., Yin N., Li H., Li D., Zhou Y., Chen S., Song L. (2025). Progress in peptide and protein therapeutics: Challenges and strategies. Acta Pharm. Sin. B.

[5] Ganz T. (2003). The role of antimicrobial peptides in innate immunity. Integr. Comp. Biol..

[6] Hancock R.E.W., Sahl H.-G. (2006). Antimicrobial and host-defense peptides as new anti-infective therapeutic strategies. Nat. Biotechnol..

[7] Tajer L., Paillart J.-C., Dib H., Sabatier J.-M., Fajloun Z., Abi Khattar Z. (2024). Molecular mechanisms of bacterial resistance to antimicrobial peptides in the modern era: An updated review. Microorganisms.

[8] Ganz T. (2003). Defensins: Antimicrobial peptides of innate immunity. Nat. Rev. Immunol..

[9] Raj P.A., Dentino A.R. (2002). Current status of defensins and their role in innate and adaptive immunity. FEMS Microbiol. Lett..

[10] Muttenthaler M., King G.F., Adams D.J., Alewood P.F. (2021). Trends in peptide drug discovery. Nat. Rev. Drug Discov..

[11] Wang C.K., Craik D.J. (2018). Designing macrocyclic disulfide-rich peptides for biotechnological applications. Nat. Chem. Biol..

[12] Tombling B.J., Wang C.K., Craik D.J. (2020). EGF-like and other disulfide-rich microdomains as therapeutic scaffolds. Angew. Chem. Int. Ed. Engl..

[13] Schwarz E. (2017). Cystine knot growth factors and their functionally versatile proregions. Biol. Chem..

[14] Wu R., Patocka J., Nepovimova E., Oleksak P., Valis M., Wu W., Kuca K. (2021). Marine invertebrate peptides: Antimicrobial peptides. Front. Microbiol..

[15] Casertano M., Menna M., Imperatore C. (2020). The ascidian-derived metabolites with antimicrobial properties. Antibiotics.

[16] Pavlicevic M., Maestri E., Marmiroli M. (2020). Marine bioactive peptides-An overview of generation, structure and application with a focus on food sources. Mar. Drugs.

[17] Arumugam V., Venkatesan M., Ramachandran S., Sundaresan U. (2018). Bioactive peptides from marine ascidians and future drug development—A review. Int. J. Pept. Res. Ther..

[18] Hansen I.K.Ø., Isaksson J., Poth A.G., Hansen K.Ø., Andersen A.J.C., Richard C.S.M., Blencke H.-M., Stensvåg K., Craik D.J., Haug T. (2020). Isolation and characterization of antimicrobial peptides with unusual disulfide connectivity from the colonial ascidian *Synoicum turgens*. Mar. Drugs.

[19] Solstad R.G., Li C., Isaksson J., Johansen J., Svenson J., Stensvåg K., Haug T. (2016). Novel antimicrobial peptides EeCentrocins 1, 2 and EeStrongylocin 2 from the edible sea urchin *Echinus esculentus* have 6-br-trp post-translational modifications. PLoS ONE.

[20] Moe M.K., Haug T., Sydnes M.O., Sperstad S.V., Li C., Vaagsfjord L.C., de la Vega E., Stensvåg K. (2018). Paralithocins, antimicrobial peptides with unusual disulfide connectivity from the red king crab, *Paralithodes camtschaticus*. J. Nat. Prod..

[21] Stensvåg K., Haug T., Sperstad S.V., Rekdal Ø., Indrevoll B., Styrvold O.B. (2008). Arasin 1, a proline–arginine-rich antimicrobial peptide isolated from the spider crab, *Hyas araneus*. Dev. Comp. Immunol..

[22] Merkler D.J. (1994). C-Terminal amidated peptides: Production by the in vitro enzymatic amidation of glycine-extended peptides and the importance of the amide to bioactivity. Enzym. Microb. Tech..

[23] Waghu F.H., Barai R.S., Idicula-Thomas S. (2016). Leveraging family-specific signatures for AMP discovery and high-throughput annotation. Sci. Rep..

[24] Wang G., Schmidt C., Li X., Wang Z. (2026). APD6: The antimicrobial peptide database is expanded to promote research and development by deploying an unprecedented information pipeline. Nucleic Acids Res..

[25] Ansel H.C., Norred W.P., Roth I.L. (1969). Antimicrobial activity of dimethyl sulfoxide against *Escherichia coli*, *Pseudomonas aeruginosa*, and *Bacillus megaterium*. J. Pharm. Sci..

[26] Albert A., Eksteen J.J., Isaksson J., Sengee M., Hansen T., Vasskog T. (2016). General approach to determine disulfide connectivity in cysteine-rich peptides by sequential alkylation on solid phase and mass spectrometry. Anal. Chem..

[27] Ovchinnikova T.V., Balandin S.V., Aleshina G.M., Tagaev A.A., Leonova Y.F., Krasnodembsky E.D., Men’shenin A.V., Kokryakov V.N. (2006). Aurelin, a novel antimicrobial peptide from jellyfish *Aurelia aurita* with structural features of defensins and channel-blocking toxins. Biochem. Bioph. Res. Co..

[28] Vidal-Dupiol J., Ladrière O., Destoumieux-Garzón D., Sautière P.-E., Meistertzheim A.-L., Tambutté E., Tambutté S., Duval D., Fouré L., Adjeroud M. (2011). Innate immune responses of a scleractinian coral to vibriosis. J. Biol. Chem..

[29] Jacob R.B., McDougal O.M. (2010). The M-superfamily of conotoxins: A review. Cell. Mol. Life Sci..

[30] Wang G., Li X., Wang Z. (2015). APD3: The antimicrobial peptide database as a tool for research and education. Nucleic Acids Res..

[31] Ma T., Liu Y., Yu B., Sun X., Yao H., Hao C., Li J., Nawaz M., Jiang X., Lao X. (2025). DRAMP 4.0: An open-access data repository dedicated to the clinical translation of antimicrobial peptides. Nucleic Acids Res..

[32] Pirtskhalava M., Amstrong A.A., Grigolava M., Chubinidze M., Alimbarashvili E., Vishnepolsky B., Gabrielian A., Rosenthal A., Hurt D.E., Tartakovsky M. (2021). DBAASP v3: Database of antimicrobial/cytotoxic activity and structure of peptides as a resource for development of new therapeutics. Nucleic Acids Res..

[33] Svangård E., Göransson U., Hocaoglu Z., Gullbo J., Larsson R., Claeson P., Bohlin L. (2004). Cytotoxic cyclotides from *Viola tricolor*. J. Nat. Prod..

[34] Göransson U., Luijendijk T., Johansson S., Bohlin L., Claeson P. (1999). Seven novel macrocyclic polypeptides from *Viola arvensis*. J. Nat. Prod..

[35] Haug T., Kjuul A.K., Stensvåg K., Sandsdalen E., Styrvold O.B. (2002). Antibacterial activity in four marine crustacean decapods. Fish Shellfish Immunol..

[36] Hansen K.Ø., Andersen J.H., Bayer A., Pandey S.K., Lorentzen M., Jørgensen K.B., Sydnes M.O., Guttormsen Y., Baumann M., Koch U. (2019). Kinase chemodiversity from the Arctic: The breitfussins. J. Med. Chem..

[37] Haug T., Stensvåg K., Olsen Ø.M., Sandsdalen E., Styrvold O.B. (2004). Antibacterial activities in various tissues of the horse mussel, *Modiolus modiolus*. J. Invertebr. Pathol..

