# Supplementary material for: Isolation and Characterization of St-CRPs: Cysteine-Rich Peptides from the Arctic Marine Ascidian Synoicum turgens
Source: Mar Drugs. 2026 May 8;24(5):168. doi: 10.3390/md24050168 (PMC13208793; doi:10.3390/md24050168)
Supplement: Supplementary file 1 [file marinedrugs-24-00168-s001.zip › marinedrugs-4281897-supplementary.pdf]

Supporting information

Isolation and characterization of St-CRPs: Cysteine-rich peptides from the Arctic marine ascidian *Synoicum turgens*

Ida K. Ø. Hansen [1,*], Philip B. Rainsford [2], Johan Isaksson [2,3], Kine Ø. Hansen [3], Klara Stensvåg [1], Anastasia Albert [4], Terje Vasskog [3] and Tor Haug [1,*]

[1] The Norwegian College of Fishery Science, Faculty of Biosciences, Fisheries and Economics, UiT The Arctic University of Norway, Breivika, N-9037 Tromsø, Norway
[2] Department of Chemistry, Faculty of Science and Technology, UiT The Arctic University of Norway, Breivika, N-9037 Tromsø, Norway
[3] Department of Pharmacy, Faculty of Health Sciences, UiT The Arctic University of Norway, Breivika, N-9037 Tromsø, Norway
[4] Norce, Siva Innovasjonssenter, Sykehusveien 21, 9019 Tromsø
* Authors to whom correspondence should be addressed.

Table of contents

Figure S1. MS spectra and deconvoluted MS spectra of St-CRP-1
Figure S2. MS spectra and deconvoluted MS spectra of St-CRP-2
Figure S3. UPLC-PDA chromatogram of St-CRP-1
Figure S4. UPLC-PDA chromatogram of St-CRP-2
Figure S5. *De novo* sequencing of St-CRP-2
Figure S6. The alkylation pattern 2xNEM + 2xNMM + 2xNCM of the acetylated $[M+2H]^{2+}$ of St-CRP-2
Table S1. Antimicrobial activity of solid phase extract (SPE) fractions and the organic extract of *S. turgens*
Table S2. Proton (^1H) NMR and chemical shift assignments for St-CRP-1
Table S3. Carbon (^{13}C) NMR and chemical shift assignments for St-CRP-1
Table S4. RMSD of top St-CRP-1 structures generated through final simulated annellation constraints

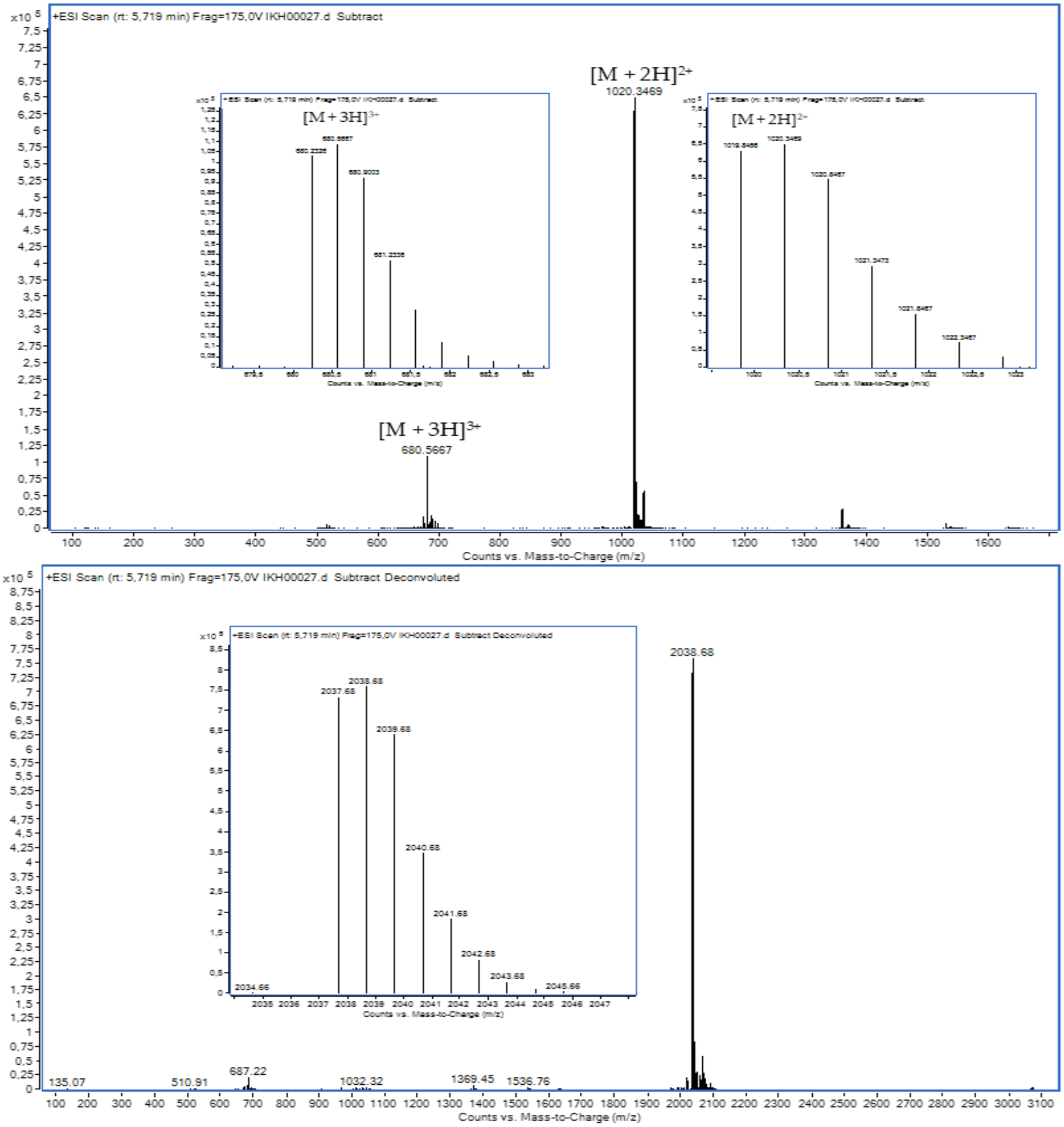

Figure S1. MS spectra of St-CRP-1 above and deconvoluted MS spectra below, both with a zoomed in spectra of the masses.

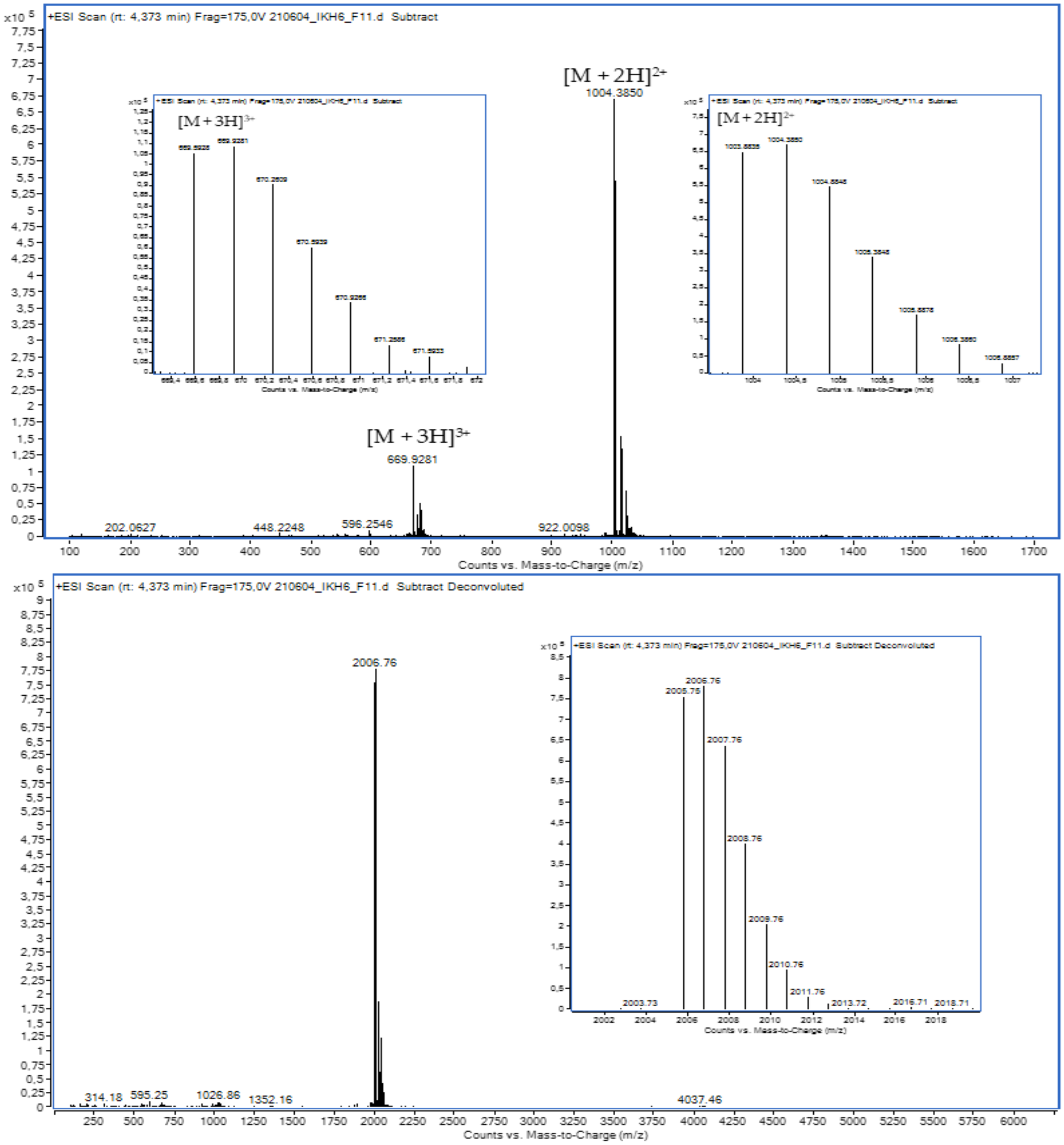

Figure S2. MS spectra of St-CRP-2 above and deconvoluted MS spectra below, both with a zoomed in spectra of the masses.

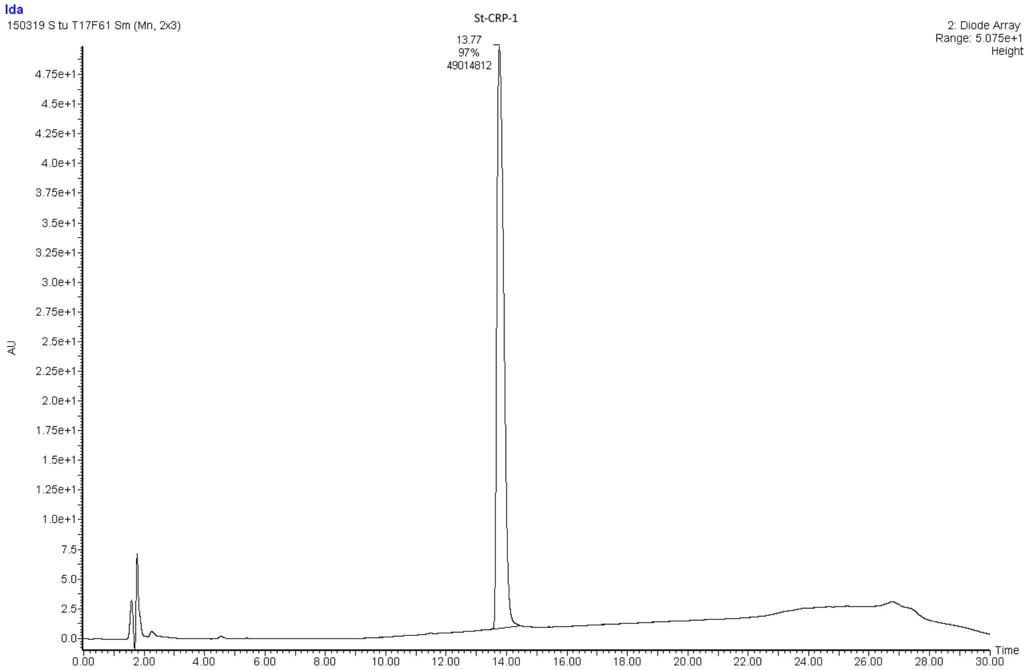

Figure S3. UPLC-PDA chromatogram to determine the purity (97%) of St-CRP-1 isolated from *S. turgens*.

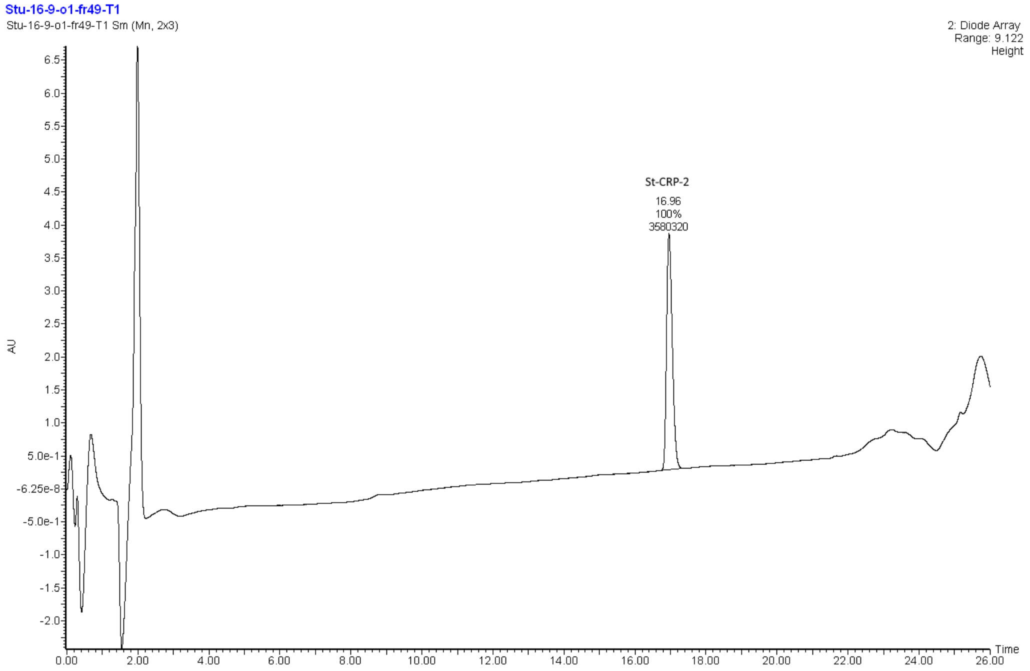

Figure S4. UPLC-PDA chromatogram to determine the purity (100%) of St-CRP-2 isolated from *S. turgens*

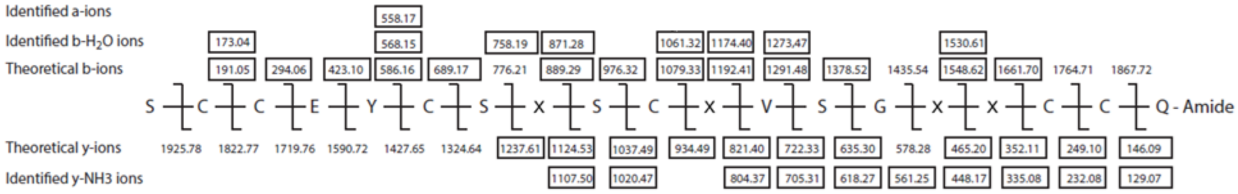

Figure S5. *De novo* sequencing of St-CRP-2 isolated from *S. turgens*, showing the identified a-, b-, b-H_2O, y- and y-NH_3 ions in frames. X = I or L. The sequencing was done on a Xevo G2-XS QToF MS (Waters).

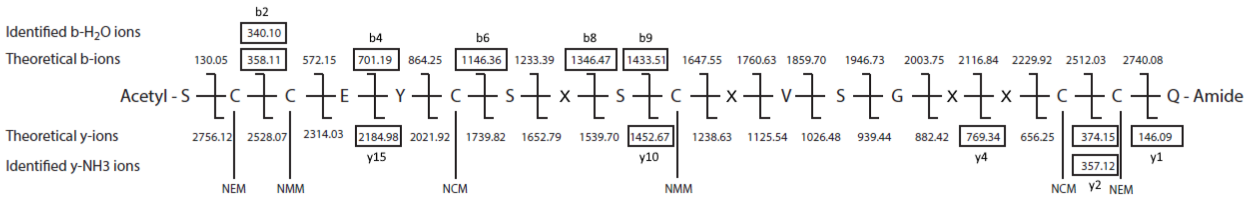

Figure S6. The alkylation pattern 2xNEM + 2xNMM + 2xNCM of the acetylated $[M+2H]^{2+}$ of St-CRP-2, isolated from *S. turgens*. The framed masses are b-, b-H_2O, y- and y-NH_3 ions identified in the MS/MS spectra. The X in the sequence is either I or L. The sequencing was done on a Xevo G2-XS QToF MS (Waters).

Table S1. Antimicrobial activity given as minimal inhibitory concentrations (MIC) of solid phase extract (SPE) fractions and the organic extract of *S. turgens*. The measurements were end point values of OD_{595} after 24 h at 35˚C. Bacterial test strains: *C. g.* - *Corynebacterium glutamicum*, *B. s.* - *Bacillus subtilis*, *S. a.* - *Staphylococcus aureus*, *E. c.* - *Escherichia coli*, *P. a.* - *Pseudomonas aeruginosa*.

	**Antimicrobial activity (MIC; mg/mL)**				
**Extract**	***C. g.***	***B. s.***	***S. a.***	***E. c.***	***P. a.***
10% SPE	1.25	5.00	5.00	10.00	5.00
20% SPE	2.50	2.50	5.00	5.00	5.00
30% SPE	0.16	0.16	2.50	5.00	5.00
40% SPE	0.04	0.08	2.50	5.00	2.50
80% SPE	0.31	0.31	2.50	5.00	2.50
Organic	2.50	2.50	10.00	>10.00	>10.00

Table S2. Proton (^1H) NMR and chemical shift assignments for St-CRP-1, isolated from *S. turgens*.

**Residue**	**N^{1}H (ppm) 600 MHz, H_2O**	**α^{1}H (ppm) 600 MHz, H_2O**	**β^{1}H (ppm) 600 MHz, H_2O**	**γ^{1}H (ppm) 600 MHz, H_2O**	**other ^{1}H (ppm) 600 MHz, H_2O**
**CYS***1*	8.80	4.349	3.038, 2.875	-	-
**CYS***2*	9.003	4.835	3.323	-	-
**ASP***3*	9.028	4.262	2.698	-	-
**GLN***4*	7.866	4.347	1.884, 1.944	2.229	6.785, 7.416
**CYS***5*	8.793	4.676	2.895, 3.018	-	-
**TYR***6*	7.581	4.596	2.812, 3.027	-	εCH: 6.772 δCH:6.926
**GLY***7*	8.686	3.703, 3.849	-	-	-
**PHE***8*	8.766	4.108	2.928, 3.182	-	εCH: 7.196 δCH: 7.233 ζCH: 7.147
**CYS***9*	8.244	3.985	3.075, 3.371	-	-
**ARG***10*	6.803	4.031	1.348, 1.632	1.439, 1.463	δCH_2: 3.060 εNH: 7.046
**LEU***11*	7.712	3.963	1.585, 1.598	1.487	δCH_3: 0.780, 0.810
**VAL***12*	7.234	4.226	2.188	0.470, 0.569	-
**ASP***13*	7.743	4.438	2.639, 4.438	-	-
**ASN***14*	8.222	5.150	2.293, 2.777	NH_2: 6.760, 7.355	-
**CYS***15*	8.656	4.814	3.228, 3.257	-	-
**CYS***16*	8.716	4.426	2.585, 3.155	-	-
**ASN***17*	8.750	4.639	2.673, 2.752	NH2: 6.792, 7.504	-
**SER***18*	8.199	4.295	3.758, 3.826	-	-

Table S3. Carbon (^{13}C) NMR and chemical shift assignments for St-CRP-1, isolated from *S. turgens*.

Residue	^{13}C (ppm) 150 MHz, H_2O	$\alpha^{13}C$ (ppm) 150 MHz, H_2O	$\beta^{13}C$ (ppm) 150 MHz, H_2O	$\gamma^{13}C$ (ppm) 150 MHz, H_2O	Other (^{13}C (ppm) 150 MHz, H_2O)
**CYS***1*	-	51.05	38.73	-	-
**CYS***2*	-	51.38	42.86	-	-
**ASP***3*	-	53.25	36.64	-	-
**GLN***4*	-	51.97	25.81	30.69	177.75
**CYS***5*	-	51.25	32.72	-	-
**TYR***6*	-	53.52	37.87	127.02	δC: 130. 58<br>εC: 115.47<br>ζC: 154.94
**GLY***7*	-	43.56	-	-	-
**PHE***8*	-	58.57	36.21	135.21	δC: 128.86<br>εC: 127.55<br>ζC: 129.01
**CYS***9*	-	56.47	41.35	-	-
**ARG***10*	-	55.58	27.39	24.30	δC: 40.625<br>ζC: 156.651
**LEU***11*	-	54.93	39.24	23.92	δC: 21.079, 21.601
**VAL***12*	-	57.88	27.79	15.74, 18.32	-
**ASP***13*	-	51.06	36.53	-	-
**ASN***14*	-	48.63	37.87	-	-
**CYS***15*	-	53.28	35.99	-	-
**CYS***16*	-	52.66	36.50	-	-
**ASN***17*	-	50.47	36.09	-	-
**SER***18*	174.32	55.59	61.02	-	-

Table S4. RMSD of top St-CRP-1 structures generated through final simulated annellation constraints

Structure Name	All atom RMSD	Backbone RMSD	Carbon RMSD	Heavy atom RMSD
SA_252	0	0	0	0
SA_463	1.215	0.323	0.217	0.675
SA_6	1.624	0.355	0.258	0.628
SA_37	1.725	0.63	0.793	0.936
SA_372	1.749	0.526	0.513	0.852
SA_487	1.776	0.527	0.546	0.816
SA_48	1.793	0.658	0.75	0.963
SA_401	1.802	0.355	0.249	0.628
SA_382	1.872	0.531	0.517	0.869
SA_202	1.874	0.602	0.73	1.003
SA_495	1.885	0.512	0.502	0.867
SA_78	1.899	0.737	0.691	1.078
SA_238	1.927	0.541	0.519	0.905
SA_20	1.967	0.924	0.917	1.182
SA_113	1.974	1.004	1.111	1.412
SA_199	1.985	0.706	0.838	0.989
SA_100	2.074	0.533	0.48	0.925
SA_11	2.101	0.781	0.704	1.028
SA_462	2.178	0.532	0.506	0.906
SA_215	2.214	0.531	0.551	0.815
SA_80	2.215	0.538	0.552	0.802
SA_221	2.239	0.612	0.668	1.025
SA_76	2.259	0.766	0.854	0.948
SA_427	2.309	0.665	0.665	0.87
SA_97	2.374	0.635	0.686	0.932
SA_226	2.433	0.732	0.815	1.03
SA_412	2.509	0.87	0.968	1.059
SA_367	3.521	2.788	3.216	2.919
SA_488	3.726	2.88	3.347	3.047
SA_338	3.989	3.211	3.657	3.364
SA_436	4.13	3.192	3.645	3.307
